# Long-term efficacy and safety of carotid artery stenting versus endarterectomy: A meta-analysis of randomized controlled trials

**DOI:** 10.1371/journal.pone.0180804

**Published:** 2017-07-14

**Authors:** Yang Li, Jing-Jing Yang, Su-Hui Zhu, Biao Xu, Lian Wang

**Affiliations:** 1 Department of Cardiology, Affiliated Drum Tower Hospital, Nanjing University School of Medicine, Nanjing China; 2 Department of Traditional Chinese Medicine, Nanjing Drum Tower Hospital, Clinical College of Traditional Chinese and Western Medicine, Nanjing University of Chinese Medicine, Nanjng, China; "INSERM", FRANCE

## Abstract

**Background:**

Many recent trials have investigated the long-term efficacy and safety of endarterectomy versus stenting in treating patients with carotid artery stenosis. We aimed to determine the long-term comparative efficacy and safety of both procedures by pooling this evidence in a meta-analysis.

**Methods:**

We searched PubMed, EMBASE, and the Cochrane Central Register of Controlled Trials for studies published until May 6, 2016. Randomized controlled trials, which reported outcomes of interest with a median follow-up of at least 4-year, were included.

**Results:**

Eight trials involving 7005 patients and 41824 patient-years of follow-up were included. In terms of the periprocedural outcomes, stenting was associated with a lower risk of myocardial infarction (OR: 0.51; 95% CI: 0.33 to 0.80; *P* = 0.003) but a higher risk of death or stroke (the composite endpoint, OR: 1.76; 95% CI: 1.38 to 2.25; *P* < 0.0001), a result that was primarily driven by minor stroke (OR: 2.19; 95% CI: 1.59 to 3.01; *P* < 0.0001), less so by periprocedural death (OR: 1.68; 95% CI: 0.82 to 3.44; *P* = 0.16) and major stroke (OR: 1.41; 95% CI: 0.95 to 2.09; *P* = 0.09). In terms of the long-term outcomes, stenting was associated with a higher risk of stroke (OR 1.45; 95% CI: 1.22 to 1.73; *P* < 0.0001) and the composite outcome of death or stroke (OR 1.25; 95% CI: 1.05 to 1.48; *P* = 0.01). No difference was found in long-term all-cause mortality between stenting and endarterectomy (OR: 1.09; 95% CI: 0.95 to 1.26; *P* = 0.21) and restenosis (OR: 1.48 (95% CI: 0.93 to 2.35; *P* = 0.10). No evidence of significant heterogeneity was found in any of the analyses.

**Conclusions:**

Carotid endarterectomy was found to be superior to stenting for short- and long-term outcomes, although endarterectomy was associated with a higher risk of periprocedural myocardial infarction. Carotid endarterectomy should be offered as the first choice for carotid stenosis at present, however, more evidence is needed because rapid progress in concurrent devices and medical treatments is being made.

## Introduction

Stroke is one of the leading causes of death and disability worldwide. Atherosclerotic stenosis of the extracranial carotid artery accounts for approximately 20% of all strokes[1]. Carotid endarterectomy is effective in lowering the risk of stroke in patients with symptomatic carotid stenosis, and it is considered to be the first-line treatment. However, since the last decade, carotid artery stenting has increasingly emerged as an alternative strategy[2]. A group of studies that presented the short–and intermediate-term (median follow-up under 4-years) comparative efficacy and safety of endarterectomy versus stenting in treating patients with carotid artery stenosis has generated largely consistent results[3]. It has been generally shown that the periprocedural risk of stroke was lower and the risk of myocardial infarction was higher for carotid endarterectomy than for stenting, while all-cause mortality and disabling stroke rates were similar. Intermediate-term outcomes were not significantly different between the two interventions[4]. These observations were consistent even after multivariable adjustments[5]. In the last 3 years, several large randomized trials with long-term follow-ups have been published, with the primary goal of comparing the use of stenting and endarterectomy and the updated long-term follow-up data of several other trials[6–10]. These important publications have made it possible to perform a meta-analysis of the long-term performance of stenting versus endarterectomy, which remains controversial because these trials did not reach consistent conclusions.

## Methods

Before performing the meta-analysis, we developed an unpublished protocol on April 29, 2016, and we followed it (S1 Appendix). Specifically, we used the Preferred Reporting Items for Systematic Reviews and Meta-analyses (S1 File) as a handbook.

### Search strategy and selection criteria

We conducted the meta-analysis in accordance with the Preferred Reporting Items for Systematic Reviews and Meta-Analyses (S1 File) guideline[11]. We sought to identify all randomized controlled trials comparing the long-term effects of stenting versus endarterectomy for carotid-artery stenosis. We searched EMBASE, the Cochrane Library Central Register of Controlled Trials (CENTRAL), and PubMed from their inceptions until May 6, 2016, using the following search terms and keywords: “Carotid stenosis”, “stent”, and “endarterectomy”(S1 Table). We also checked the reference lists of the identified reports and several relevant reviews.

### Study selection

Two investigators (YL and JY) independently assessed the study eligibility. The following inclusion criteria were followed: the included studies 1) were RCTs that performed head-to-head comparisons between stenting and endarterectomy in treating patients with carotid artery stenosis, regardless of whether they were symptomatic or asymptomatic patients or a mixed population, 2) contained at least 20 patients and reported the outcomes of interest with a median follow-up of at least 4-year, and 3) were published in peer-review journals. For the studies that satisfied the basic inclusion criteria, the following exclusion criteria were applied before the full texts were reviewed: 1) non-randomized prospective trials, 2) retrospective trials, 3) observational analysis, 4) systemic reviews and meta-analyses, 5) old versions of included RCTs, 6) a median follow-up period of less than 4 years, 7) full text was not available, and 8) non-human studies. Discrepancies, if any, were resolved by consensus with a third independent investigator (SZ).

### Outcome measurements

The periprocedural outcomes among these studies included stroke, death, myocardial infarction, cranial nerve palsy and hematoma. The postprocedural outcomes included death, stroke and restenosis. In detail, stroke was subdivided into major stroke and minor stroke. The major stroke group included fatal and disabling strokes, which included contralateral, ipsilateral, and bilateral strokes. The minor stroke group included non-disabling strokes of the same variety. Because the available data from the included studies were limited, we analyzed only partial endpoints. The primary endpoints were the long-term risk of stroke and a composite endpoint of death, ipsilateral stroke or periprocedural stroke. Other endpoints included long-term death, ipsilateral stroke, and restenosis. The definitions for the outcomes of interest were adopted from the original articles[6, 7].

### Data extraction and quality assessment

Pre-specified data elements were extracted by two investigators (YL and JY), who followed a standardized data extraction form, which included the baseline characteristics, length of medium follow-up, enrolling period, sample size, proportion of symptomatic and asymptomatic patients, patients finished procedure, inclusion and exclusion criteria, and embolic protection device use. For the measured outcomes, we extracted all data endpoints from each study and classified all data, according to our needs. In addition, two investigators independently evaluated the potential risk of bias in each trial, according to the Cochrane Collaboration guidelines[12], which include random sequence generation (selection bias), allocation concealment (selection bias), blinding of the participants and personnel (performance bias), blinding of the outcomes assessment (detection bias), incomplete outcome data (attrition bias), selective reporting (reporting bias) and other sources of bias. In cases of disagreement, the third independent investigator judged (SZ).

### Statistical analysis

Odds ratios (OR) and corresponding 95% confidence intervals (CIs) s were used to estimate the relative treatment effects for dichotomous outcomes[13]. The Higgins’s and Thompson’s *I*^*2*^ statistics were used to evaluate statistical heterogeneity between the studies[14]. In case there was no significant heterogeneity, we used fixed-effect models (Mantel-Haenszel method)[15], otherwise random-effect models (DerSimonian and Laird methods) were used[16]. When there were zero events in one of the treatment groups, we estimated the treatment effect by adding 0.5 to each cell of the 2×2 table. To analyze long-term outcomes, we used the rate of outcome per 100 patient-years (PYs) rather than the number of events because the former incorporated the duration of the trials, and there was considerable variability in the follow-up periods for each of these trials. Publication bias was evaluated using funnel plots, Begg and Egger tests[17, 18]. The sensitivity analysis was performed by excluding one trial at a time. We performed meta-regression analysis to estimate the effects of covariates on stroke and the composite endpoint, including the patient type (symptomatic or asymptomatic) and the use of embolic-protection device. All analyses were performed using RevMan 5.3 software (The Cochrane Collaboration).

## Results

### Search results and study characteristics

The flow diagram of the study is shown in S1 Fig. Of the 978 articles initially identified, 8 trials involving 7005 patients and 41824 PYs follow-ups fulfilled the inclusion criteria and were included in the analysis [6–10, 19–31]: 3869 patients (22800 PYs follow-up) received stenting and 3136 patients (19024 PYs follow-up) received endarterectomy. The medium follow-up time was at least 4 years. The mean patient age ranged from 66 to 70 years. The percentage of males in the stenting group ranged from 61.2% to 80%, and the percentage of male in endarterectomy group ranged from 56.9% to 90% (Table 1 and S2 Table). The overall clinical follow-up rate was 95.0% for the stenting group and 96.9% for the endarterectomy group. The Asymptomatic Carotid Trial (ACT) I trial only targeted patients with asymptomatic severe carotid-artery stenosis, while the other trials included patients with symptomatic carotid stenosis or a mixed population of symptomatic and asymptomatic patients. The Carotid and Vertebral Artery Transluminal Angioplasty Study (CAVATAS) trial used balloon angioplasty and stenting in the endovascular group[19, 24], while the other 7 trials used stenting only. The quality of the randomized trials is reported in S3 Table. The risk of bias was low in most trials, except for the Endarterectomy Versus Angioplasty in Patients with Symptomatic Severe Carotid Stenosis (EVA-3S) trial, which stopped for safety and futility reasons and was considered to have unclear bias.

**Table 1:** Selected baseline characteristics of the included randomized controlled trials.

Trial	Enrolling period	Publication year	Follow-up, years	No. of patients (CAS/CEA)	Asymptomatic	Symptomatic	Age, years (CAS/CEA)	Procedures (CAS/CEA)	EPD (CAS/CEA)
ACT I	2005–2013	2016	5	1089/364	1453	0	67.7/67.9	1032/343	Yes
CREST	2000–2008	2016	7.4	1271/1251	1176	1326	68.9/69.2	1262/1240	Depends
ICSS	2001–2011	2015	4.2	855/858	0	1713	70/70	752/811	Depends
EVA-3S	2000–2005	2014	7.1	265/262	0	527	70.2/69.1	247/257	Depends
BACASS	1998–2002	2008	4	10/10	0	20	70	10/10	Unclear
Markus, et al	1999–2002	2008	5.4	43/44	0	87	67.9/68.4	42/42	No
Kentucky, et al	1998–2002	2014	> 10	95/94	85	104	66.4/69.6	90/83	No
CAVATAS	1992–1997	2009	5	251/253	50	454	68/68	251/253	Unclear

CAS: Carotid Artery Stenting; CEA: Carotid Endarterectomy; EPD: Embolic Protection Device; ACT I: Asymptomatic Carotid Trial I; CREST: Carotid Revascularization Endarterectomy vs. Stenting Trial; ICSS: International Carotid Stenting Study; EVA-3S: Endarterectomy Versus Angioplasty in Patients with Symptomatic Severe Carotid Stenosis; BACASS: Basel Carotid Artery Stent Study; CAVATAS: Carotid and Vertebral Artery Transluminal Angioplasty Study.

### Periprocedural outcomes

**Fig 1:**
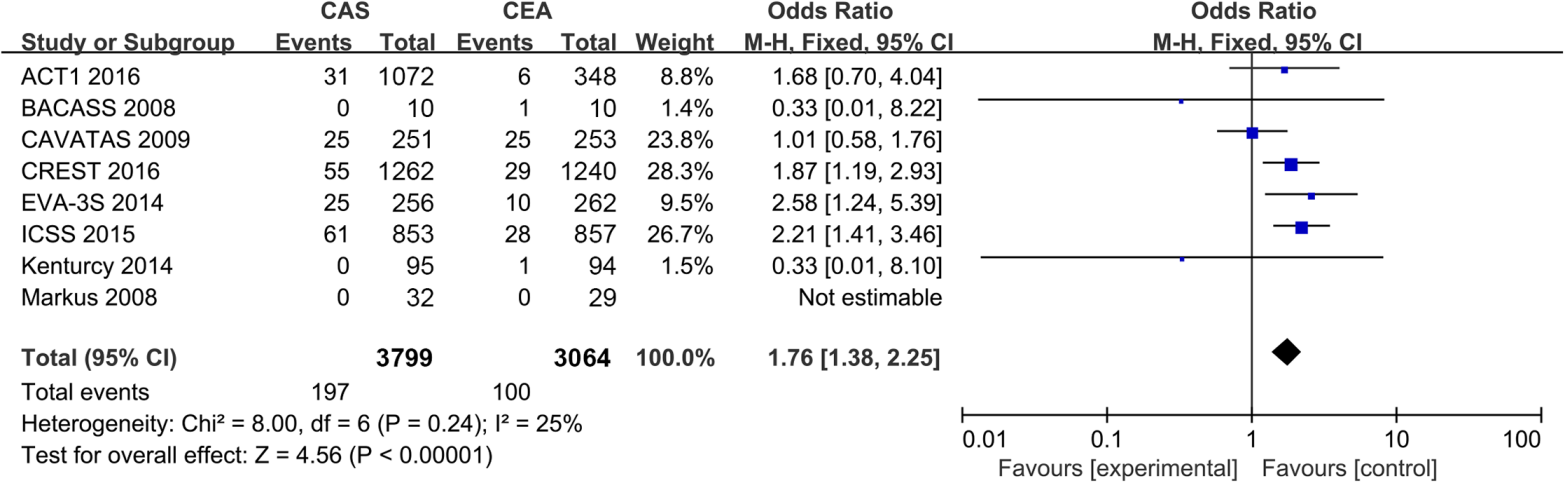
The cumulative incidence of the composite outcome of death or stroke. CAS: Carotid Artery Stenting; CEA: Carotid Endarterectomy; ACT I: Asymptomatic Carotid Trial I; CREST: Carotid Revascularization Endarterectomy vs. Stenting Trial; ICSS: International Carotid Stenting Study; EVA-3S: Endarterectomy Versus Angioplasty in Patients with Symptomatic Severe Carotid Stenosis; BACASS: Basel Carotid Artery Stent Study; CAVATAS: Carotid and Vertebral Artery Transluminal Angioplasty Study; and Odd Ratio: adopted per 100 patient-years odd ratio.

A periprocedural outcome was defined as an endpoint that occurred during the 30 days following the intervention or 36 days after the randomization if the procedure was not performed within 30 days after the randomization. The pooling data showed statistically significantly decreased odds of periprocedural myocardial infarction with stenting (OR: 0.52; 95% CI: 0.33 to 0.81; *P* = 0.004) (S2 Fig). No evidence of significant heterogeneity was found (*I*^*2*^ = 0%). The sensitivity analyses did not generate inconsistent results. Stenting was associated with a significantly higher incidence of the composite outcome of death or stroke (OR: 1.76; 95% CI: 1.38 to 2.25; *P* < 0.0001) (Fig 1), which was primarily driven by a higher rate of minor stroke (OR: 2.19; 95% CI: 1.59 to 3.01; *P* < 0.0001) (S3 Fig) and less driven by higher incidences of periprocedural death (OR: 1.68; 95% CI: 0.82 to 3.44; *P* = 0.16) (S4 Fig) and major stroke (OR: 1.41; 95% CI: 0.95 to 2.09; *P* = 0.09) (S5 Fig).

### Long-term outcomes

**Fig 2:**
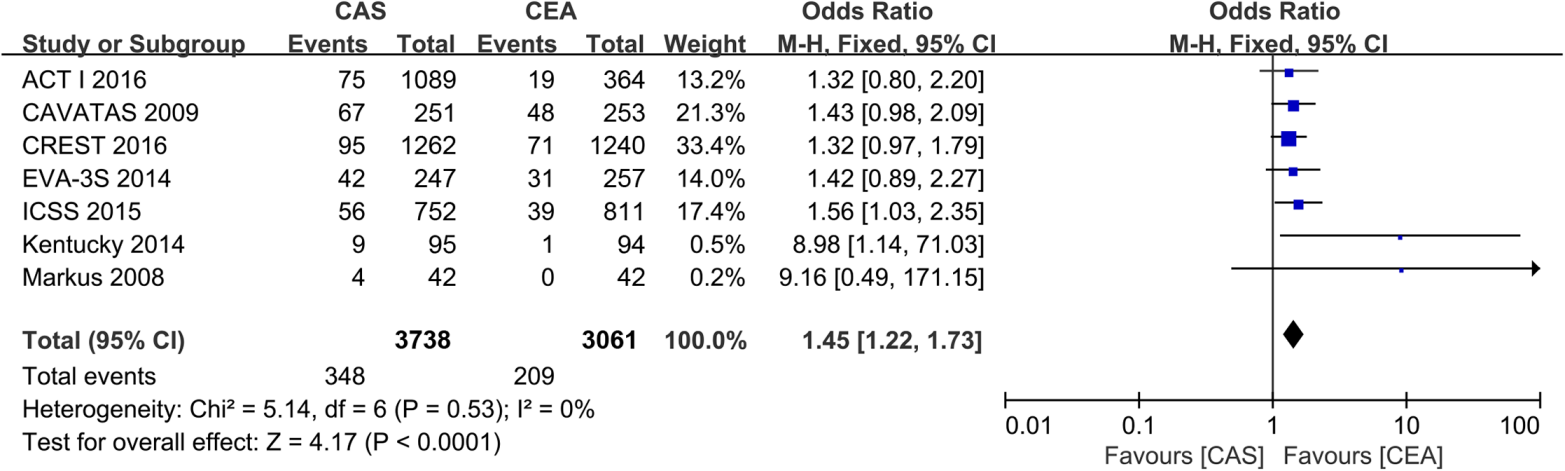
The cumulative incidence of stroke. CAS: Carotid Artery Stenting; CEA: Carotid Endarterectomy; ACT I: Asymptomatic Carotid Trial I; CREST: Carotid Revascularization Endarterectomy vs. Stenting Trial; ICSS: International Carotid Stenting Study; EVA-3S: Endarterectomy Versus Angioplasty in Patients with Symptomatic Severe Carotid Stenosis; CAVATAS: Carotid and Vertebral Artery Transluminal Angioplasty Study; Odd Ratio: adopted per 100 patient-years odd ratio.

Long-term outcomes were defined as the postprocedural events that occurred from the beginning of the periprocedural period to the last time of follow-up. Seven trials, including 6799 patients and 40787 PYs follow-ups, contributed to the analysis of stroke, which included contralateral stroke and ipsilateral stroke. The meta-analysis showed that stenting was associated with a significantly higher risk of stroke (event rate 9.3% vs 6.8, OR 1.45, 95% CI: 1.22 to 1.73, *P* < 0.0001) (Fig 2).

**Fig 3:**
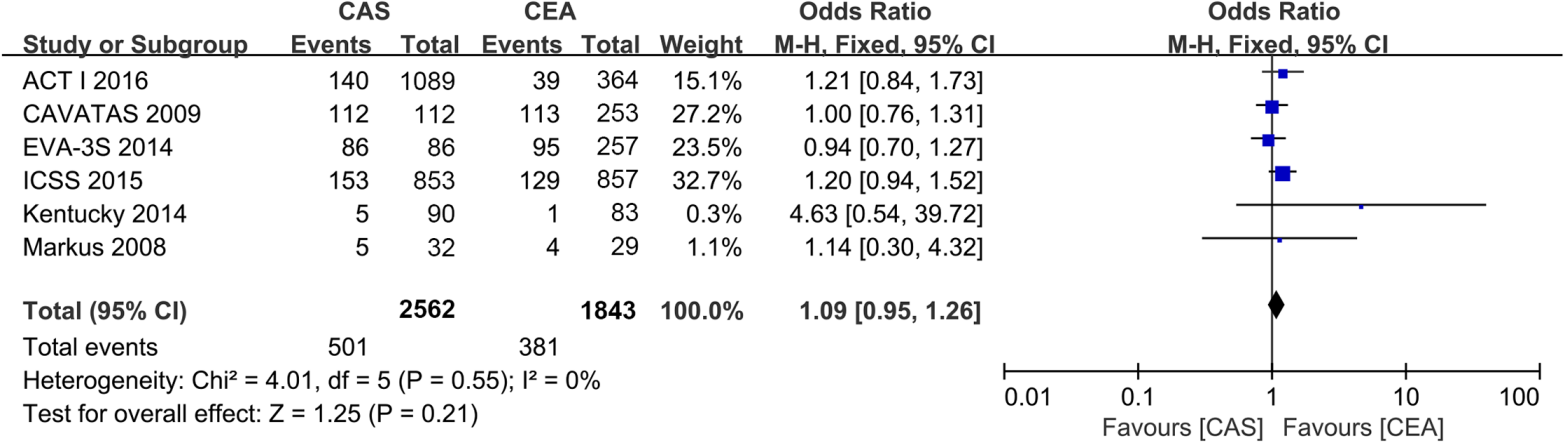
The cumulative incidence of long-term all-cause mortality. CAS: Carotid Artery Stenting; CEA: Carotid Endarterectomy; ACT I: Asymptomatic Carotid Trial I; ICSS: International Carotid Stenting Study; EVA-3S: Endarterectomy Versus Angioplasty in Patients with Symptomatic Severe Carotid Stenosis; CAVATAS: Carotid and Vertebral Artery Transluminal Angioplasty Study; Odd Ratio: adopted per 100 PYs odd ratio.

A total of 501 of 2562 patients with stenting and 381 of 1843 patients with endarterectomy died during the long-term follow-ups. The long-term all-cause mortality rate did not differ between stenting and endarterectomy (OR: 1.09; 95% CI: 0.95 to 1.26; *P* = 0.21) (Fig 3). Similarly, there was no statistically significant difference in the ipsilateral stroke rate between stenting and endarterectomy (207 events in 40333 PYs of follow-up; OR: 1.04; 95% CI: 0.79 to 1.37; *P* = 0.80) (S6 Fig).

**Fig 4:**
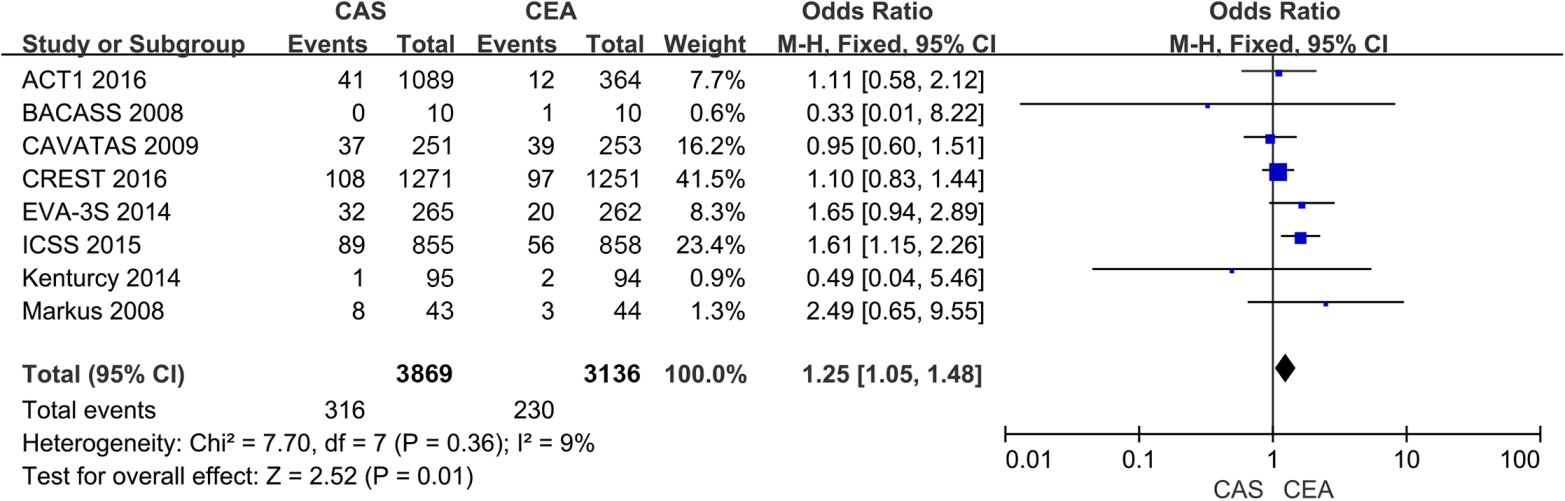
The cumulative incidence of the composite endpoint of death, ipsilateral stroke or periprocedural stroke. CAS: Carotid Artery Stenting; CEA: Carotid Endarterectomy; ACT I: Asymptomatic Carotid Trial I; CREST: Carotid Revascularization Endarterectomy vs. Stenting Trial; ICSS: International Carotid Stenting Study; EVA-3S: Endarterectomy Versus Angioplasty in Patients with Symptomatic Severe Carotid Stenosis; BACASS: Basel Carotid Artery Stent Study; CAVATAS: Carotid and Vertebral Artery Transluminal Angioplasty Study; Odd Ratio: adopted per 100 patient-years odd ratio.

Eight trials contributed to the analysis of the composite endpoint of death, ipsilateral stroke or periprocedural stroke, with 316 patients in the stenting group and 230 patients in the endarterectomy group reaching this endpoint. Stenting significantly increased the incidence of the composite outcome compared with endarterectomy (OR 1.25; 95% CI: 1.05 to 1.48; *P* = 0.01) (Fig 4).

**Fig 5:**
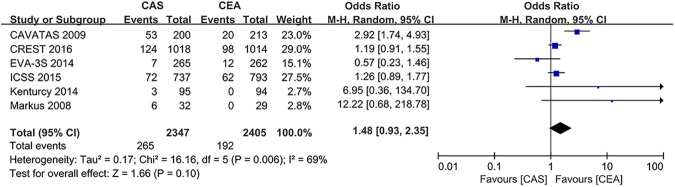
The cumulative incidence of restenosis. CAS: Carotid Artery Stenting; CEA: Carotid Endarterectomy; CREST: Carotid Revascularization Endarterectomy vs. Stenting Trial; ICSS: International Carotid Stenting Study; EVA-3S: Endarterectomy Versus Angioplasty in Patients with Symptomatic Severe Carotid Stenosis; CAVATAS: Carotid and Vertebral Artery Transluminal Angioplasty Study; Odd Ratio: adopted per 100 patient-years odd ratio.

The overall incidence of restenosis in the stenting group was 11.3% (265 events in 2347 patients over 15099 PYs follow-up), whereas it was 8.0% in the endarterectomy group (192 events in 2405 patients over 14857 PYs of follow-up). Endarterectomy was associated with a numerically higher restenosis rate, with a combined OR of 1.48 (95% CI: 0.93 to 2.35; *P* = 0.10) (Fig 5).

We did not detect significant heterogeneity across these trials in any of the analyses, and no significant publication bias was found. Sensitivity analyses confirmed the consistency of our main findings. Meta-regression analysis did not show that the two study-level variables, patient type (*P* for interaction = 0.56) or use of an embolic-protection device (*P* for interaction = 0.32) had an effect on either stroke or the composite endpoint for stenting versus endarterectomy.

## Discussion

Following our previous review protocol, we wanted to analyze as many endpoints as possible. Finally, we only analyzed the reported data from the including studies. In this system review and meta-analysis of 7005 patients and 41824 patient-years of follow-up in 8 randomized trials, we showed that stenting was associated with a significantly higher risk of long-term stroke (by 45%), the composite endpoint of ipsilateral stroke or periprocedural stroke or death (by 25%), and the periprocedural risk of the composite outcome of death or stroke (by 76%), but it significantly reduced the risk of periprocedural myocardial infarction (by 49%) compared to endarterectomy. Meanwhile, stenting was associated with a numerically higher incidence of long-term restenosis, periprocedural death and major stroke. Between these two arms, the accumulative incidences of long-term ipsilateral stroke showed no significant difference (with 113 events per 21901 PYs in CAS arm and 94 events per 18432 PYs, OR: 1.04 [0.79–1.37], *P* = 0.80) and long-term mortality (501 events per 13110 PYs in CAS arm and 381 events per 9496 PYs, OR: 1.09 [0.95–1.26], *P* = 0.21). The meta-regression analyses, based on study population and the use of embolic-protection devices, did not reveal any interaction between these covariates and the endpoints. Note that heterogeneity across trials in all comparisons did not reach the level of statistical significance, which reinforced the consistency of our findings.

When we searched PubMed for other relevant meta-analyses, we found 4 studies that provided long-term data analyses[32–36]. Our findings were similar to those of these meta-analyses, which also showed higher risks for long-term risk of stroke or the composite endpoint of stroke or death in patients receiving carotid stenting. However, our findings significantly extended the work in the following aspects. First, our analysis was the largest meta-analysis, and it provided the most convincing evidence, which included the recently published Asymptomatic Carotid Trial (ACT) I trial[8] (1453 patients), the updated 10-year-follow-up results of the Carotid Revascularization Endarterectomy versus Stenting Trial (CREST)[7] (2502 patients), 4-year-follow-up results of the International Carotid Stenting Study (ICSS) trial[6] (1713 patients), and 7-year-follow-up results of the EVA-3S trial [9](527 patients). Vincent and colleagues have not included the ACT I trial and updated CREST trial[7, 8]. Zhang and colleagues have analyzed both RCTs and observational studies, but they did not include most of the recent large RCTs[33]. Luebke and colleagues have not included the ACT I trial[34]. A Cochrane meta-analysis by Coward, L. J and his colleagues provided 30-day and 1-year follow-up result[35]. They did not include most of the recent RCTs[6–9, 27, 28], and they updated some RCTs[10, 19]. Two of those included studies ended at an early stage, with no long-term follow-up data. The Long-term Results of Carotid Stenting versus Endarterectomy in High-Risk Patients trial (SAPPHIRE) from the Cochrane meta-analysis included only high-risk patients[37], which was not consistent with our research purposes because we wanted to explore the impact of patients who meet North American Symptomatic Carotid Endarterectomy Trial criteria. The most recent meta-analysis by Howard, G had described an association between age and risk of stroke or death from carotid endarterectomy and carotid stenting[36]. The patient-level study only included patients with symptomatic stenosis and showed that CEA was superior to CAS for patients ranging in age from 70 to 74 years and older. The difference was primarily attributed to increasing periprocedural stroke risk in the CAS arm. Simultaneously, this study showed that age had no effect between CAS and CEA during the postprocedural period. We needed more studies to determine the risk that affected the superiority of CEA in the long-term result. Second, we only included trials with long-term follow-up data (i.e., a median follow-up period longer than 4 years), but the studies of Vincent and Zhang, Luebke included intermediate- and long-term follow-up data (mixed analysis), making their results difficult to interpret[32–34]. For the same reason, we did not include the results of the Stent-Protected Angioplasty versus Carotid Endarterectomy (SPACE) study, which ended after a median 2-year follow-up because of a lack of funding[38]. Finally, the mean follow-up periods differed in all studies. Increasing the mean follow-up time may have impacted the cumulative endpoint event. Therefore, we used the rate of outcome per 100 patient-years rather than the number of events to incorporate the duration of the trials (with considerable variability) and, thus, provided the most precise estimation, while all other meta-analyses did not consider the duration, which could have resulted in estimation bias.

All patients from both treatment groups were primarily symptomatic (60.5%), and only three RCTs contained both asymptomatic and symptomatic patients. Subgroup analyses of symptomatic and asymptomatic patients were only performed in the CREST study, which showed no significant differences based on the treatment groups in the primary composite endpoint or in the rate of postprocedural stroke over the 10-year follow-up. Because we lacked original data, we did not perform subgroup analyses of asymptomatic and symptomatic patients in this meta-analysis. More RCTs, containing both asymptomatic and symptomatic patients, were needed to determine better treatment strategies for different patients.

When restenosis was identified, we realized that different studies defined restenosis in a similar manner. Restenosis, in the included six studies[6, 10, 27, 29–31], was defined as any severe stenosis ≥70% or occlusion of the carotid artery detected at any stage during the follow-up after completed treatment. Data from two other studies were not published. Duplex ultrasound velocity tended to be a well-established and validated method of estimating stenosis in carotid arteries[39]. Duplex ultrasound examinations were performed 1 month, 6 months, and 1 year after treatment and annually thereafter in all studies. However, the heterogeneity came from two areas. First, there were differences in thee diagnostic criteria. Two RCTs (CAVATAS and ICSS) used peak systolic velocity >2.1 m/s for both groups as a diagnostic criterion. Another two RCTs (CREST and Kentucky) performed the diagnosis using a peak systolic velocity of at least 3·0 m/s. Note that ultrasound may overestimate the degree of stenosis, particularly when a stent is present; EVA-3S used peak systolic velocities of 2.1 m/s in patients treated with CEA and of velocities of 3 m/s for those treated with CAS, which made the diagnosis of restenosis higher in the CEA arm than in the CAS arm (with 7 events per 1882 PYs in CAS arm and 12 events per 1860 PYs, OR: 0.57 [0.23–1.46], *P* = 0.26). The Markus study did not mention its diagnostic criteria. Second, ultrasound examinations were performed by different doctors in different centers. Ultrasound examinations in two RCTs (CAVATAS and EVA-3S) were performed in many centers, and in other RCTs (CREST, ICSS, Markus and Kentucky) they were performed in one center. To obtain more objective conclusions, the researchers must perform future studies to define certain diagnostic criteria.

In our analysis, the only endpoint that favored stenting was periprocedural myocardial infarction. The following two reasons might partially explain why endarterectomy was associated with a higher incidence of periprocedural myocardial infarction. First, combined antiplatelet therapy, which is the standard therapy used to prevent cardiovascular disease, is less commonly used in patients scheduled for endarterectomy (compared to stenting) to decrease the risk of bleeding. Second, cervical incision during the endarterectomy procedure induces local inflammation and produces proinflammatory cytokines, which could lead to thrombus formation[40]. Note that most of the pivotal carotid endarterectomy trials were designed and began enrollment over a decade ago, when medical treatment for atherosclerosis was far from optimized. Considering the rapid advances in the medical treatment of atherosclerosis in recent years, it is probable that endarterectomy combined with current medical therapy would result in lower occurrence of periprocedural myocardial infarction, even though direct comparisons of endarterectomy with medical therapy are warranted to determine whether an individual revascularization/non-revascularization strategy or a combined strategy of revascularization and medical management should be adapted. In terms of endarterectomy, improvements in preoperative cardiac evaluation and anesthesia might also reduce the risk of perioperative myocardial infarction[41].

Our findings suggested that endarterectomy has more favorable periprocedural and long-term stroke outcomes, as well as composite outcomes (i.e., stroke or death). Therefore, endarterectomy should be preferred to manage carotid stenosis. However, it should not be ignored that carotid artery stenting is a relatively new technique, and the associated complications may be attributed to a learning curve[4]. Recent advances in the use of emboli protection devices, dual antiplatelet therapy, and mesh-covered stents, and improvements in procedure experience may decrease the risk of strokes following stent implantation[7]. Further studies are needed to address the relative efficacy and safety of stenting versus endarterectomy or medical treatment in the future. The ongoing ACST-2 (Asymptomatic Carotid Surgery Trial-2) trial (ClinicalTrials.gov: NCT00883402) with 3600 patients, and the CREST-2[42, 43] (Carotid Revascularization and Medical Management for Asymptomatic Carotid Stenosis Trial) trial (Clinical Trials.gov: NCT02089217) with 2480 patients were designed to answer these questions.

Several limitations should be acknowledged in our study. First, our meta-analysis was based on study-level data instead of patient-level data. Second, due to the limited number of trials included, it is underpowered to perform subgroup analysis for certain variables that could affect the outcomes, such as age, gender, patient type (symptomatic or asymptomatic), use of an embolic-protection device, etc. We did perform meta-regression analysis for several variables, and we did not find significant interactions; however, this finding should only be regarded as hypothetical. Third, our conclusions were based on evidence that was primarily from symptomatic patients. Only the ACT I trial focused on asymptomatic patients with carotid artery stenosis. The ACT I trial did not find significant differences in terms of all individual outcomes or composite outcomes. Therefore, the conclusion in our study may not be simply generalizable to asymptomatic patients. Fourth, 8 of the included RCTs did not specify the definitions of cranial nerve palsy and hematoma, therefore, we were unable to analyze these two events.

## Conclusion

In our analysis of over 7000 patients with carotid stenosis and over 41000 patient-years of follow-up, carotid stenting was associated with a lower risk of periprocedural myocardial infarction, but it increased the risk of periprocedural death or stroke, as well as the long-term risk for stroke, restenosis, and the composite endpoint of stroke or death. Our data suggest that carotid endarterectomy should be offered as the first choice for carotid stenosis at present, but more evidence is needed to reevaluate the comparative efficacy and safety of both techniques because rapid progress is being made in the development of devices and medical treatments.

## Supporting information

S1 Appendix The study protocol. (DOCX)

S2 Appendix The certification of editing. (PDF)

S1 Fig A flow diagram of the study. (TIF)

S2 Fig The cumulative incidence of periprocedural myocardial infarction. CAS: Carotid Artery Stenting; CEA: Carotid Endarterectomy; CREST: Carotid Revascularization Endarterectomy vs. Stenting Trial; ICSS: International Carotid Stenting Study; EVA-3S: Endarterectomy Versus Angioplasty in Patients with Symptomatic Severe Carotid Stenosis; CAVATAS: Carotid and Vertebral Artery Transluminal Angioplasty Study; Odd Ratio: Adopted per 100 patient-years odd ratio.
(TIF)

S3 Fig The cumulative incidence of the minor stroke. CAS: Carotid Artery Stenting; CEA: Carotid Endarterectomy; ACT I: Asymptomatic Carotid Trial I; CREST: Carotid Revascularization Endarterectomy vs. Stenting Trial; ICSS: International Carotid Stenting Study; EVA-3S: Endarterectomy Versus Angioplasty in Patients with Symptomatic Severe Carotid Stenosis; BACASS: Basel Carotid Artery Stent Study; CAVATAS: Carotid and Vertebral Artery Transluminal Angioplasty Study; Odd Ratio: Adopted per 100 patient-years odd ratio.
(TIF)

S4 Fig The cumulative incidence of periprocedural death. CAS: Carotid Artery Stenting; CEA: Carotid Endarterectomy; ACT I: Asymptomatic Carotid Trial I; CREST: Carotid Revascularization Endarterectomy vs. Stenting Trial; ICSS: International Carotid Stenting Study; EVA-3S: Endarterectomy Versus Angioplasty in Patients with Symptomatic Severe Carotid Stenosis; BACASS: Basel Carotid Artery Stent Study; CAVATAS: Carotid and Vertebral Artery Transluminal Angioplasty Study; Odd Ratio: Adopted per 100 patient-years odd ratio.
(TIF)

S5 Fig The cumulative incidence of major stroke. CAS: Carotid Artery Stenting; CEA: Carotid Endarterectomy; ACT I: Asymptomatic Carotid Trial I; CREST: Carotid Revascularization Endarterectomy vs. Stenting Trial; ICSS: International Carotid Stenting Study; EVA-3S: Endarterectomy Versus Angioplasty in Patients with Symptomatic Severe Carotid Stenosis; BACASS: Basel Carotid Artery Stent Study; CAVATAS: Carotid and Vertebral Artery Transluminal Angioplasty Study; Odd Ratio: Adopted per 100 patient-years odd ratio.
(TIF)

S6 Fig The cumulative incidence of ipsilateral stroke. CAS: Carotid Artery Stenting; CEA: Carotid Endarterectomy; ACT I: Asymptomatic Carotid Trial I; CREST: Carotid Revascularization Endarterectomy vs. Stenting Trial; ICSS: International Carotid Stenting Study; EVA-3S: Endarterectomy Versus Angioplasty in Patients with Symptomatic Severe Carotid Stenosis; CAVATAS: Carotid and Vertebral Artery Transluminal Angioplasty Study; Odd Ratio: Adopted per 100 patient-years odd ratio.
(TIF)

S1 Table Search strategies for trials comparing carotid artery stenting to carotid endarterectomy. (PDF)

S2 Table Other characteristics of the included randomized controlled trials. CAD: Coronary artery disease; CAS: Carotid Artery Stenting; CEA: Carotid Endarterectomy; ACT I: Asymptomatic Carotid Trial I; CREST: Carotid Revascularization Endarterectomy vs. Stenting Trial; ICSS: International Carotid Stenting Study; EVA-3S: Endarterectomy Versus Angioplasty in Patients with Symptomatic Severe Carotid Stenosis; BACASS: Basel Carotid Artery Stent Study; CAVATAS: Carotid and Vertebral Artery Transluminal Angioplasty Study.
(PDF)

S3 Table Risk of bias of the included randomized controlled trials. CAS: Carotid Artery Stenting; CEA: Carotid Endarterectomy; ACT I: Asymptomatic Carotid Trial I; CREST: Carotid Revascularization Endarterectomy vs. Stenting Trial; ICSS: International Carotid Stenting Study; EVA-3S: Endarterectomy Versus Angioplasty in Patients with Symptomatic Severe Carotid Stenosis; BACASS: Basel Carotid Artery Stent Study; CAVATAS: Carotid and Vertebral Artery Transluminal Angioplasty Study.
(PDF)

S1 File PRISMA 2009 checklist. (DOC)
