# Correction: Long-term efficacy and safety of carotid artery stenting versus endarterectomy: A meta-analysis of randomized controlled trials

**DOI:** 10.1371/journal.pone.0202932

**Published:** 2018-08-20

**Authors:** Yang Li, Jing-Jing Yang, Su-Hui Zhu, Biao Xu, Lian Wang

The following information is missing from the Funding section: This work was supported in part by Six Talent Peaks Project of Jiangsu Province, China (2016-WSN-157). The funders had no role in study design, data collection and analysis, decision to publish, or preparation of the manuscript.
