# Supplementary material for: Long-term efficacy and safety of carotid artery stenting versus endarterectomy: A meta-analysis of randomized controlled trials
Source: PLoS One. 2017 Jul 14;12(7):e0180804. doi: 10.1371/journal.pone.0180804 (PMC5510818; doi:10.1371/journal.pone.0180804)
Supplement: S1 Appendix — (DOCX) [file pone.0180804.s001.docx]

**Long-term Efficacy and Safety of Carotid Artery Stenting versus Endarterectomy: a Meta-Analysis of Randomized Controlled Trials**

Yang Li, Jing-Jing Yang, Su-Hui Zhu, Biao Xu, Lian Wang.

## Review question(s)

Comparing long-term efficacy and safety which strategy is superior to patients with carotid artery stenosis? endarterectomy or stenting.

## Searches

We will search the following databases; PubMed, Embase, and The Cochrane Collection.

Inclusion criteria: 1) be randomized controlled trials which made head-to-head comparisons of stenting with endarterectomy for treating patients with carotid artery stenosis, regardless they were symptomatic or asymptomatic patients or a mixed population; 2) contain at least 20 patients and report outcomes of interest with an median follow-up at least 4-year.

Exclusion criteria: 1) Non-randomized prospective trials;2) Retrospective trials;3) Observational trials;4) Systemic reviews and meta-analyses; 5) Old version of included RCTs;6) the median followed-up less than 4 years;7) Full text is not available;8) Not human studies.

All studies published before the 06.05.2016 which meet the inclusion criteria will be eligible for inclusion, but only full text studies in English will be included.

The reference lists from the included studies will be reviewed to find additional studies. In addition, the references from excluded reviews and editorials will also be reviewed to identify studies that were not found in the initial search.

## Types of study to be included

Randomized controlled trials only.

## Condition or domain being studied

Advancement in treatment of carotid artery stenosis.

## Participants

Human research only

## Intervention

Stenting for carotid artery stenosis.

## Control

Endarterectomy for carotid artery stenosis.

## Outcome(s)

Periprocedural outcomes included stroke, death, myocardial infarction, cranial nerve palsy and hematoma.

Postprocedural outcomes included death, stroke and restenosis.

## Data extraction

Two reviewers will independently screen studies by titles and abstracts, and this will be followed by full text reading. Any discrepancies will be resolved by a reexamination of the studies until a consensus is reached. If they will not reach a consensus, there will be a third reviewer to examine again to judge. Data from the studies, including: authors, year of publication, study design, type and number of patients and outcome will be recorded.

Two authors will independently extract data from the included studies, and any discrepancies will be resolved by a reexamination of the studies until a consensus is reached. If they will not reach a consensus, there will be a third reviewer to examine again to judge.

## Risk of bias (quality) assessment

The quality of the studies will be assessed using the methodological index of the Cochrane Collaboration’s tool for assessing risk of bias.

## Strategy for data synthesis

A narrative synthesis of the findings from the included studies will be conducted around the development of advancement in treatment of carotid artery stenosis.

A quantitative analysis will be conducted if an adequate number of sufficiently homogenous studies are available.

## Analysis of subgroups

None.

## Contact details for further information

Yang Li.

Department of Cardiology, Affiliated Drum Tower Hospital, Nanjing University School of Medicine, Nanjing China.

Lynju2010@126.com.

## Organizational affiliation of the review

Department of Cardiology, Affiliated Drum Tower Hospital, Nanjing University School of Medicine, 321 Zhongshan Road, 210008 Nanjing, Jiangsu Province, China.

## Review team

Yang Li, Nanjing University School of Medicine;

Jing-Jing Yang, Nanjing University of Chinese Medicine;

Su-Hui Zhu, Nanjing University School of Medicine;

Biao Xu, Nanjing University School of Medicine;

Lian Wang, Nanjing University School of Medicine;

## Anticipated or actual start date

1 May 2016

## Anticipated completion date

30 October 2016

## Funding sources

None

## Conflicts of interest

None know

## Language

English

## Country

China
