# Supplementary material for: Long-term efficacy and safety of carotid artery stenting versus endarterectomy: A meta-analysis of randomized controlled trials
Source: PLoS One. 2017 Jul 14;12(7):e0180804. doi: 10.1371/journal.pone.0180804 (PMC5510818; doi:10.1371/journal.pone.0180804)
Supplement: S2 Appendix — (PDF) [file pone.0180804.s002.pdf]

PAID ON 04/27/2017 10:39 PM

American Journal Experts

Send to:
李洋
18120178276
南京鼓楼医院
中山路321号南京鼓楼医院心血管内科
南京市, 江苏省
China, 210008

American Journal Experts
601 West Main Street, Suite 102
Durham, NC 27701, United States
Phone: 1-919-704-4253
Fax: 1-919-287-2439
http://www.aje.com
Email: support@aje.com
Tax ID: 412141424

Invoice

Receipt code: **PREPRI-C3B-0427223802**
Authors: Yang Li, Jing-Jing Yang, Su-Hui Zhu, Biao Xu, Lian Wang
Title: Long-term Efficacy and Safety of Carotid Artery Stenting versus Endarterectomy: a Meta-Analysis of Randomized Controlled Trials
Submission date: April 27 2017, 10:39 pm

Invoice date	Description	Length	Time	Area of study	Price
April 27, 2017	Standard Editing	Standard (3501 - 6000 words)	5 days	Cardiac & Cardiovascular Systems	$303.00
				Bank credits	$35.00
				Word count under	- $35.00
				Promotional Code Welcome20	- $20.00
				Fapiao surcharge	$22.64
				Remaining balance	$-35.00

PAYMENT METHOD(S): Alipay
TERMS: Net 30 days. Online order
NOTES:
This invoice has already been paid, and is for your internal records only. Thank you for choosing American Journal Experts.

$35.00 was added to your AJE Bank because the word count and/or timeline of your manuscript was updated.
