# Supplementary figures and images for: Long-term efficacy and safety of carotid artery stenting versus endarterectomy: A meta-analysis of randomized controlled trials
Source: PLoS One. 2017 Jul 14;12(7):e0180804. doi: 10.1371/journal.pone.0180804 (PMC5510818; doi:10.1371/journal.pone.0180804)

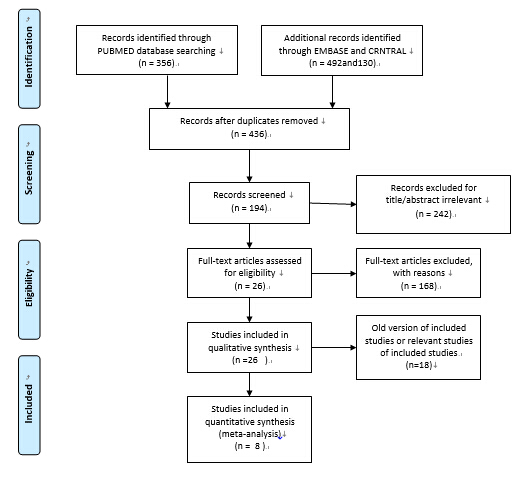

Supplement: S1 Fig — (TIF) [file pone.0180804.s003.tif]

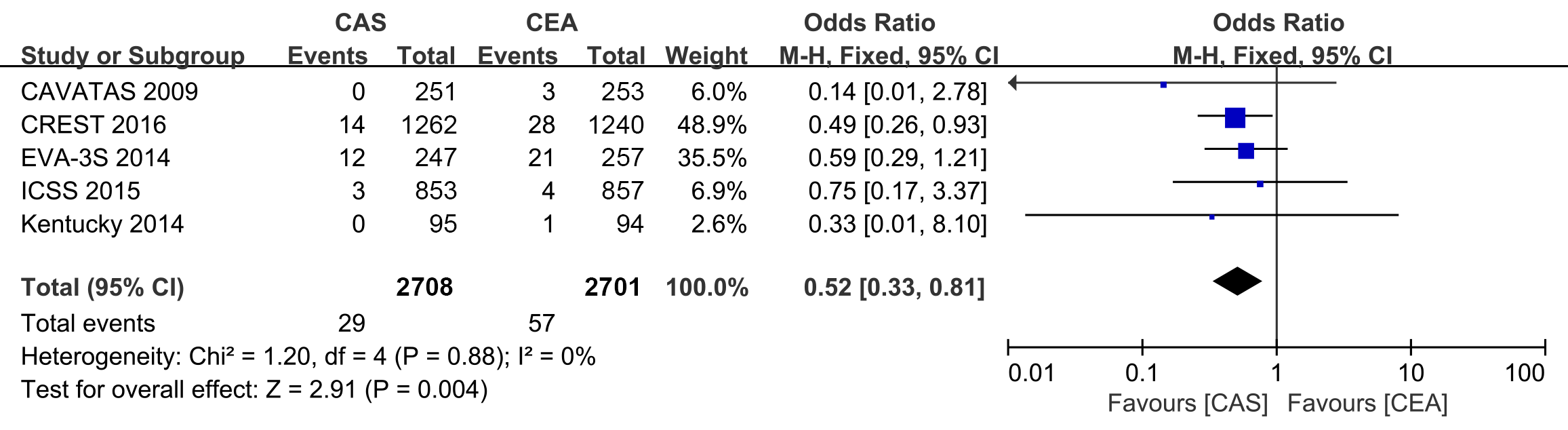

Supplement: S2 Fig — CAS: Carotid Artery Stenting; CEA: Carotid Endarterectomy; CREST: Carotid Revascularization Endarterectomy vs. Stenting Trial; ICSS: International Carotid Stenting Study; EVA-3S: Endarterectomy Versus Angioplasty in Patients with Symptomatic Severe Carotid Stenosis; CAVATAS: Carotid and Vertebral Artery Transluminal Angioplasty Study; Odd Ratio: Adopted per 100 patient-years odd ratio. (TIF) [file pone.0180804.s004.tif]

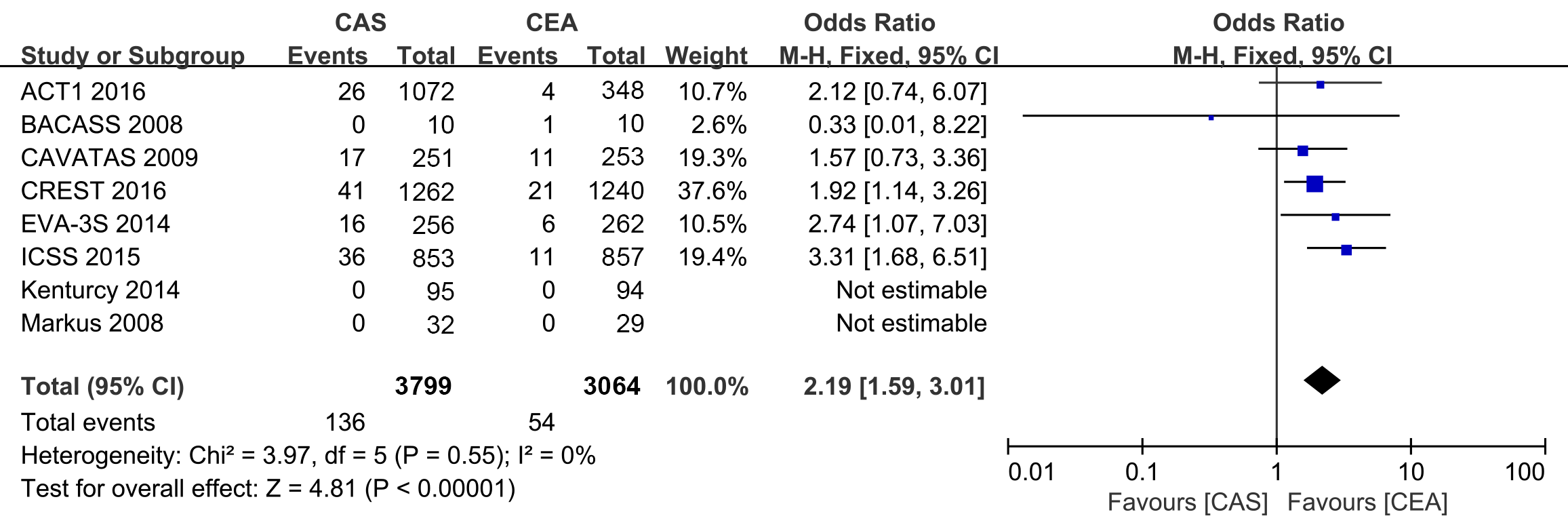

Supplement: S3 Fig — CAS: Carotid Artery Stenting; CEA: Carotid Endarterectomy; ACT I: Asymptomatic Carotid Trial I; CREST: Carotid Revascularization Endarterectomy vs. Stenting Trial; ICSS: International Carotid Stenting Study; EVA-3S: Endarterectomy Versus Angioplasty in Patients with Symptomatic Severe Carotid Stenosis; BACASS: Basel Carotid Artery Stent Study; CAVATAS: Carotid and Vertebral Artery Transluminal Angioplasty Study; Odd Ratio: Adopted per 100 patient-years odd ratio. (TIF) [file pone.0180804.s005.tif]

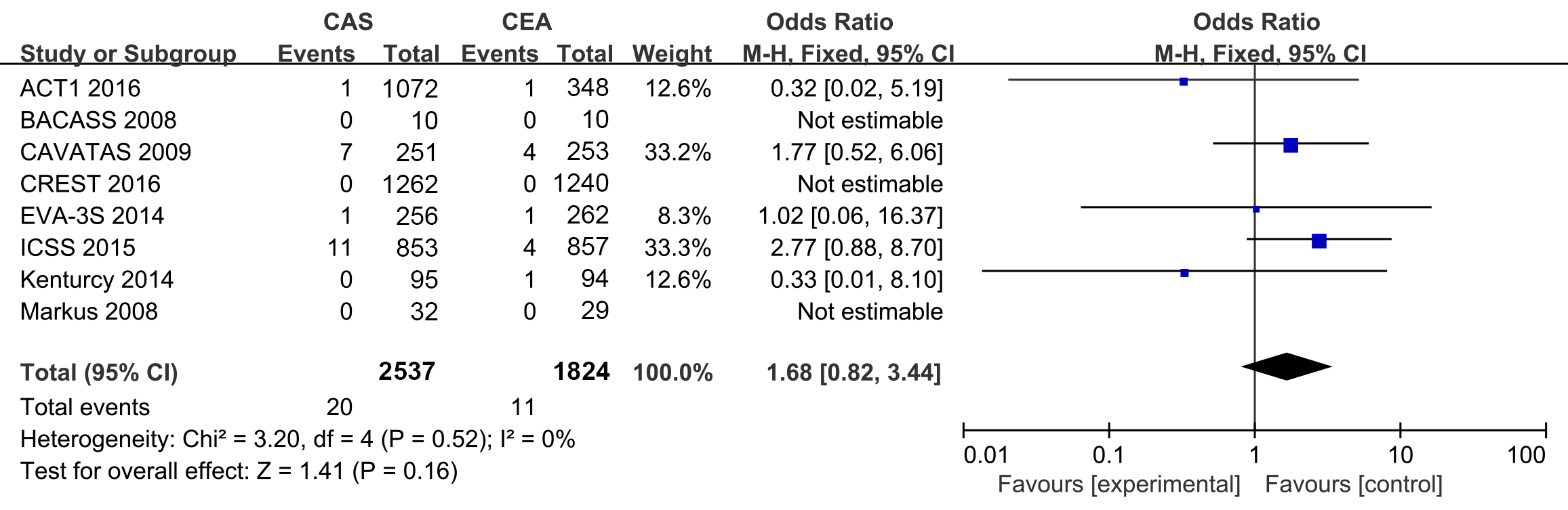

Supplement: S4 Fig — CAS: Carotid Artery Stenting; CEA: Carotid Endarterectomy; ACT I: Asymptomatic Carotid Trial I; CREST: Carotid Revascularization Endarterectomy vs. Stenting Trial; ICSS: International Carotid Stenting Study; EVA-3S: Endarterectomy Versus Angioplasty in Patients with Symptomatic Severe Carotid Stenosis; BACASS: Basel Carotid Artery Stent Study; CAVATAS: Carotid and Vertebral Artery Transluminal Angioplasty Study; Odd Ratio: Adopted per 100 patient-years odd ratio. (TIF) [file pone.0180804.s006.tif]

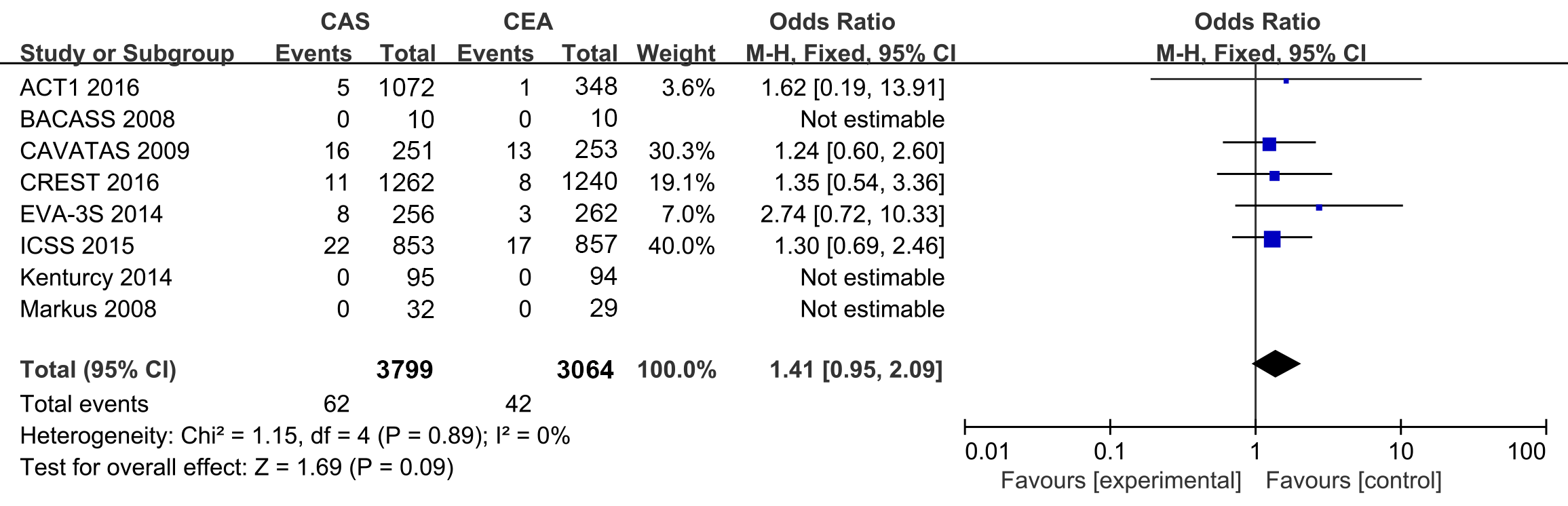

Supplement: S5 Fig — CAS: Carotid Artery Stenting; CEA: Carotid Endarterectomy; ACT I: Asymptomatic Carotid Trial I; CREST: Carotid Revascularization Endarterectomy vs. Stenting Trial; ICSS: International Carotid Stenting Study; EVA-3S: Endarterectomy Versus Angioplasty in Patients with Symptomatic Severe Carotid Stenosis; BACASS: Basel Carotid Artery Stent Study; CAVATAS: Carotid and Vertebral Artery Transluminal Angioplasty Study; Odd Ratio: Adopted per 100 patient-years odd ratio. (TIF) [file pone.0180804.s007.tif]

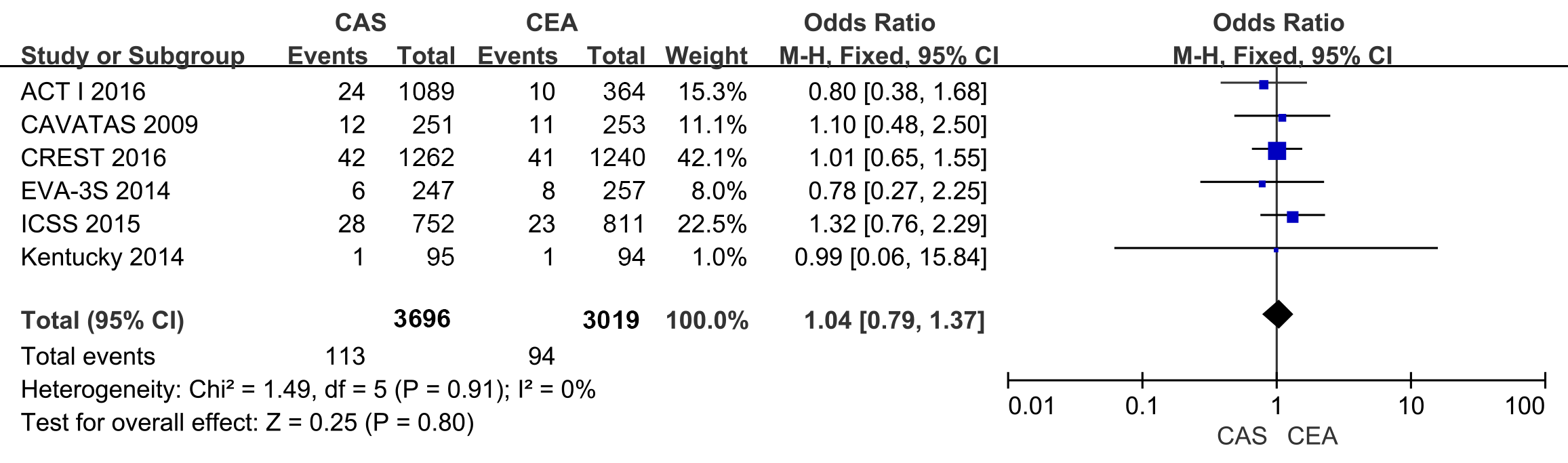

Supplement: S6 Fig — CAS: Carotid Artery Stenting; CEA: Carotid Endarterectomy; ACT I: Asymptomatic Carotid Trial I; CREST: Carotid Revascularization Endarterectomy vs. Stenting Trial; ICSS: International Carotid Stenting Study; EVA-3S: Endarterectomy Versus Angioplasty in Patients with Symptomatic Severe Carotid Stenosis; CAVATAS: Carotid and Vertebral Artery Transluminal Angioplasty Study; Odd Ratio: Adopted per 100 patient-years odd ratio. (TIF) [file pone.0180804.s008.tif]
