# Supplementary material for: Long-term efficacy and safety of carotid artery stenting versus endarterectomy: A meta-analysis of randomized controlled trials
Source: PLoS One. 2017 Jul 14;12(7):e0180804. doi: 10.1371/journal.pone.0180804 (PMC5510818; doi:10.1371/journal.pone.0180804)
Supplement: S1 Table — (PDF) [file pone.0180804.s009.pdf]

S1.Table. Searching strategies for trials comparing carotid artery stenting to carotid endarterectomy (until May 6, 2016).

Search Tool	Description	Result
**PUBMED**	((((carotid stenosis[MeSH Terms]) or ((carotid*[Title/Abstract]) and stenos*[Title/Abstract]))) and ((stents[MeSH Terms]) or ((carotid*[Title/Abstract]) and stent*[Title/Abstract])) and (((carotid endarterectomy[MeSH Terms]) or ((carotid*[Title/Abstract]) and endarterectom*[Title/Abstract]))) and (((randomized controlled trial[pt]) or (controlled clinical trial[pt]) or (randomized[tiab] or randomized[tiab]) or (placebo[tiab]) or (drug therapy[sh]) or (randomly[tiab]) or (trial[tiab]) or (groups[tiab])) not (animals[mh])) and limited to English only	356
**EMBASE**	((exp carotid artery/ and exp stenosis/) or (carotid*.mp. and stenosis.mp.)) and (((carotid artery disease/ or carotid*.mp.) and (stent*.mp. or exp stent/)) or (exp carotid artery stenting/)) and (((exp carotid artery disease/ or carotid*.mp.) and (endartarectom*.mp. or exp endartarectomy/)) or (exp carotid endarterectomy/)) and (crossover-procedure/ or double-blind procedure/ or randomized controlled trial/ or single-blind procedure/ or (random* or factorial* or crossover* or cross over* or placebo* or (doubl* adj blind*) or (singl* adj blind*) or assign* or allocat* or volunteer*).tw.) and limited to English only	492
**Cochrane**	(MeSH descriptor: [Carotid Stenosis] explode all trees) or (carotid*:ti,ab,kn (Word variations have been searched)) and (stenos*:ti,ab,kn (Word variations have been searched)) and ((MeSH descriptor: [Carotid Artery Diseases] explode all trees) and (MeSH descriptor:[Stents] explode all trees)) or ((carotid*:ti,ab,kn (Word variations have been searched)) and (stent*:ti,ab,kn (Word variations have been searched))) and ((MeSH descriptor: [Carotid Artery Diseases] explode all trees) and (MeSH descriptor:[Endarterectomy] explode all trees)) or ((carotid*:ti,ab,kn (Word variations have been searched)) and (endarterectom*:ti,ab,kn (Word variations have been searched))) or (MeSH descriptor: [Endarterectomy, Carotid] explode all trees) and limited to randomized trials	130
