# Supplementary material for: Long-term efficacy and safety of carotid artery stenting versus endarterectomy: A meta-analysis of randomized controlled trials
Source: PLoS One. 2017 Jul 14;12(7):e0180804. doi: 10.1371/journal.pone.0180804 (PMC5510818; doi:10.1371/journal.pone.0180804)
Supplement: S2 Table — CAD: Coronary artery disease; CAS: Carotid Artery Stenting; CEA: Carotid Endarterectomy; ACT I: Asymptomatic Carotid Trial I; CREST: Carotid Revascularization Endarterectomy vs. Stenting Trial; ICSS: International Carotid Stenting Study; EVA-3S: Endarterectomy Versus Angioplasty in Patients with Symptomatic Severe Carotid Stenosis; BACASS: Basel Carotid Artery Stent Study; CAVATAS: Carotid and Vertebral Artery Transluminal Angioplasty Study. (PDF) [file pone.0180804.s010.pdf]

S1 Table. Other characteristics of included randomized controlled trials.

Trial	Male, %		Hypertension, %		Hyperlipidemia, %		Smoking, %		Diabetes, %		CHD, %	
	CAS	CEA	CAS	CEA	CAS	CEA	CAS	CEA	CAS	CEA	CAS	CEA
ACT I	61.2	56.9	90.6	89.6	90	87.9	73.7	71.2	35.6	32.4	53.4	51.1
CREST	65.2	66.5	85.8	86.1	82.9	85.8	26.4	26.1	30.6	30.4	42.4	45
ICSS	70.5	70.7	69	70	61	66	72	71	22	22	18	18
EVA-3S	77.9	72.8	72	72	56	58	23	25	26	22	13	11
BACASS	80.0	90.0	70	80	70	60	50	60	30	30	20	40
Markus, et al	69.3	70.4	79.1	77.3	51.2	52.3	44.2	40.9	44.2	34.1	41.9	45.5
Kentucky, et al	NA	NA	84.9	94.1	64.2	47.1	71.7	78.4	35.8	23.5	73.6	60.8
CAVATAS	NA	NA	53	58	34	32	77	78	14	13	19	17

CAD: Coronary artery disease; CAS: Carotid Artery Stenting; CEA: Carotid Endarterectomy; ACT I: Asymptomatic Carotid Trial I; CREST: Carotid Revascularization Endarterectomy vs. Stenting Trial; ICSS: International Carotid Stenting Study; EVA-3S: Endarterectomy Versus Angioplasty in Patients with Symptomatic Severe Carotid Stenosis; BACASS: BAsel Carotid Artery Stent Study; CAVATAS: Carotid and Vertebral Artery Transluminal Angioplasty Study.
