# Supplementary material for: Long-term efficacy and safety of carotid artery stenting versus endarterectomy: A meta-analysis of randomized controlled trials
Source: PLoS One. 2017 Jul 14;12(7):e0180804. doi: 10.1371/journal.pone.0180804 (PMC5510818; doi:10.1371/journal.pone.0180804)
Supplement: S3 Table — CAS: Carotid Artery Stenting; CEA: Carotid Endarterectomy; ACT I: Asymptomatic Carotid Trial I; CREST: Carotid Revascularization Endarterectomy vs. Stenting Trial; ICSS: International Carotid Stenting Study; EVA-3S: Endarterectomy Versus Angioplasty in Patients with Symptomatic Severe Carotid Stenosis; BACASS: Basel Carotid Artery Stent Study; CAVATAS: Carotid and Vertebral Artery Transluminal Angioplasty Study. (PDF) [file pone.0180804.s011.pdf]

S2 Table. Risk of bias of included randomized controlled trials

Trial	Year	Random sequence generation (selection bias)	Allocation concealment (selection bias)	Blinding of participants and personnel (performance bias)	Blinding of outcomes assessment (detection bias)	Incomplete outcome data (attrition bias)	Selective reporting (reporting bias)	Other bias
ACT1	2016	Low risk	Low risk	Low risk	Low risk	Low risk	Low risk	Low risk
BACASS	2008	Unclear	Unclear	Low risk	Unclear	Low risk	Low risk	Unclear
CAVATAS	2009	Low risk	Low risk	Low risk	Low risk	Low risk	Low risk	Low risk
CREST	2016	Low risk	Low risk	Low risk	Low risk	Low risk	Low risk	Low risk
EVA-3S	2014	Low risk	Unclear	Low risk	Low risk	Low risk	Low risk	Unclear
ICSS	2015	Low risk	Low risk	Low risk	Low risk	Low risk	Low risk	Low risk
Kentucky, et al	2014	Unclear	Low risk	Unclear	Low risk	Low risk	Low risk	Low risk
Markus, et al	2008	Unclear	Unclear	Unclear	Low risk	Low risk	Low risk	Unclear

CAS: Carotid Artery Stenting; CEA: Carotid Endarterectomy; ACT I: Asymptomatic Carotid Trial I; CREST: Carotid Revascularization Endarterectomy vs. Stenting Trial; ICSS: International Carotid Stenting Study; EVA-3S: Endarterectomy Versus Angioplasty in Patients with Symptomatic Severe Carotid Stenosis; BACASS: BAsel Carotid Artery Stent Study; CAVATAS: Carotid and Vertebral Artery Transluminal Angioplasty Study. Unclear: The article provides information cannot be judged
